# A Variant in the Neuropeptide Receptor *npr-1* is a Major Determinant of *Caenorhabditis elegans* Growth and Physiology

**DOI:** 10.1371/journal.pgen.1004156

**Published:** 2014-02-27

**Authors:** Erik C. Andersen, Joshua S. Bloom, Justin P. Gerke, Leonid Kruglyak

**Affiliations:** 1 Lewis-Sigler Institute for Integrative Genomics, Princeton University, Princeton, New Jersey, United States of America; 2 Department of Molecular Biology, Princeton University, Princeton, New Jersey, United States of America; 3 Departments of Human Genetics and Biological Chemistry, David Geffen School of Medicine, University of California Los Angeles, Los Angeles, California, United States of America; 4 Howard Hughes Medical Institute, Chevy Chase, Maryland, United States of America; The University of North Carolina at Chapel Hill, United States of America

## Abstract

The mechanistic basis for how genetic variants cause differences in phenotypic traits is often elusive. We identified a quantitative trait locus in *Caenorhabditis elegans* that affects three seemingly unrelated phenotypic traits: lifetime fecundity, adult body size, and susceptibility to the human pathogen *Staphyloccus aureus*. We found a QTL for all three traits arises from variation in the neuropeptide receptor gene *npr-1*. Moreover, we found that variation in *npr-1* is also responsible for differences in 247 gene expression traits. Variation in *npr-1* is known to determine whether animals disperse throughout a bacterial lawn or aggregate at the edges of the lawn. We found that the allele that leads to aggregation is associated with reduced growth and reproductive output. The altered gene expression pattern caused by this allele suggests that the aggregation behavior might cause a weak starvation state, which is known to reduce growth rate and fecundity. Importantly, we show that variation in *npr-1* causes each of these phenotypic differences through behavioral avoidance of ambient oxygen concentrations. These results suggest that variation in *npr-1* has broad pleiotropic effects mediated by altered exposure to bacterial food.

## Author Summary

Using the nematode roundworm *Caenorhabditis elegans*, we identified differences in lifetime fecundity, adult body size, and susceptibility to the human pathogen *Staphyloccus aureus* between the laboratory strain (N2) from Bristol, England and a wild strain (CB4856) from Hawaii, USA. Using linkage mapping and other genetic tests, we found a QTL for all three traits arises from variation in the neuropeptide receptor gene *npr-1*. Moreover, we found that variation in *npr-1* is also responsive for differences in 247 gene expression traits. Variation in *npr-1* is known to determine whether animals disperse throughout a bacterial lawn or aggregate at the edges of the lawn. We found that the allele that leads to aggregation is associated with reduced growth and reproductive output likely caused by a weak chronic starvation state. These results suggest that variation in *npr-1* has broad effects on the phenotype of an organism mediated by altered exposure to bacterial food.

## Introduction

In recent years, quantitative genetic approaches in the nematode *Caenorhabditis elegans* have identified quantitative trait genes (QTGs) for a diverse set of phenotypes [1]–[13]. While a few studies have employed genome-wide association mapping in wild isolates [4], [14], [15], most have relied on linkage mapping in recombinant inbred lines from crosses between the common laboratory strain, N2, and a wild strain from Hawaii [16]. N2, also called “Bristol” after the site of its original collection from the wild, was isolated around 1951. For the next 18 years, this strain was propagated in the laboratory for hundreds of generations, which likely led to adaptation to the laboratory environment [7]. Therefore, the use of the Bristol strain as a parent in recombinant inbred line panels may be expected to result in the identification of variants selected during laboratory adaptation. Indeed, the use of a laboratory-adapted strain in mapping panels has led to the identification of pleiotropic laboratory-derived variants in the classic model organisms *S. cerevisiae* [17], [18] and *A. thaliana* [19]. Variants specific to laboratory strains may be expected to show large and widespread phenotypic effects when traits related to fitness are measured in the same laboratory environment in which they were selected.

Here, we take advantage of an advanced intercross recombinant inbred line (RIAIL) panel from a cross between N2 and the Hawaii strain CB4856 [15] to identify the genetic basis of differences in lifetime fecundity, adult body size, susceptibility to an opportunistic human pathogen, and hundreds of gene expression traits. We use nearly isogenic lines (NILs) and loss-of-function alleles to show that the causative gene underlying a major QTL for all these traits is the neuropeptide receptor *npr-1*, which harbors a laboratory-derived variant in the Bristol strain [7], [15].

The version of the neuropeptide receptor NPR-1 found in the Hawaii strain responds to a single neuropeptide named FLP-21 [20]. By contrast, the variant in NPR-1 found in the Bristol strain causes an abnormal gain-of-function phenotype in which NPR-1 responds to FLP-21 and an additional unrelated neuropeptide FLP-18. McGrath and colleagues found that the variant of *npr-1* found in the Bristol strain was selected sometime during laboratory propagation of the Bristol strain before long-term storage in 1969 [7]. All known wild nematode species, including all *bone fide* wild *C. elegans* strains, have the Hawaii version of NPR-1 that responds to FLP-21 [15], [21]. The abnormal response to the unrelated neuropeptide found only in the Bristol strain creates a hyperactive neural circuit through the RMG interneuron [22]. This abnormal circuit alters a wide variety of behaviors, including aggregation, aerotaxis, ethanol tolerance, hermaphrodite leaving, responses to nematode pheromones, sleep-like states, and avoidance of carbon dioxide, heat, and pathogen [5], [7], [9], [21]–[28]. We show that the physiological consequences of *npr-1* variation are mediated by alteration of aerotaxis behavior. In this behavior, Bristol animals no longer seek lower oxygen levels when consuming bacterial food [24]. By contrast, normal wild-type *C. elegans* strains (like Hawaii) aggregate in the low oxygen regions at the edge of the bacterial lawn. This differential exposure to food during growth and development ultimately leads to differences in adult body size, fecundity, and physiology of *C. elegans*. These results illustrate the wide-ranging consequences of some laboratory-derived alleles and expand our understanding of the molecular basis and mechanism of pleiotropy.

## Results

### The Bristol and Hawaii strains differ in lifetime fecundity, adult body size, and response to pathogens

**Figure 1:**
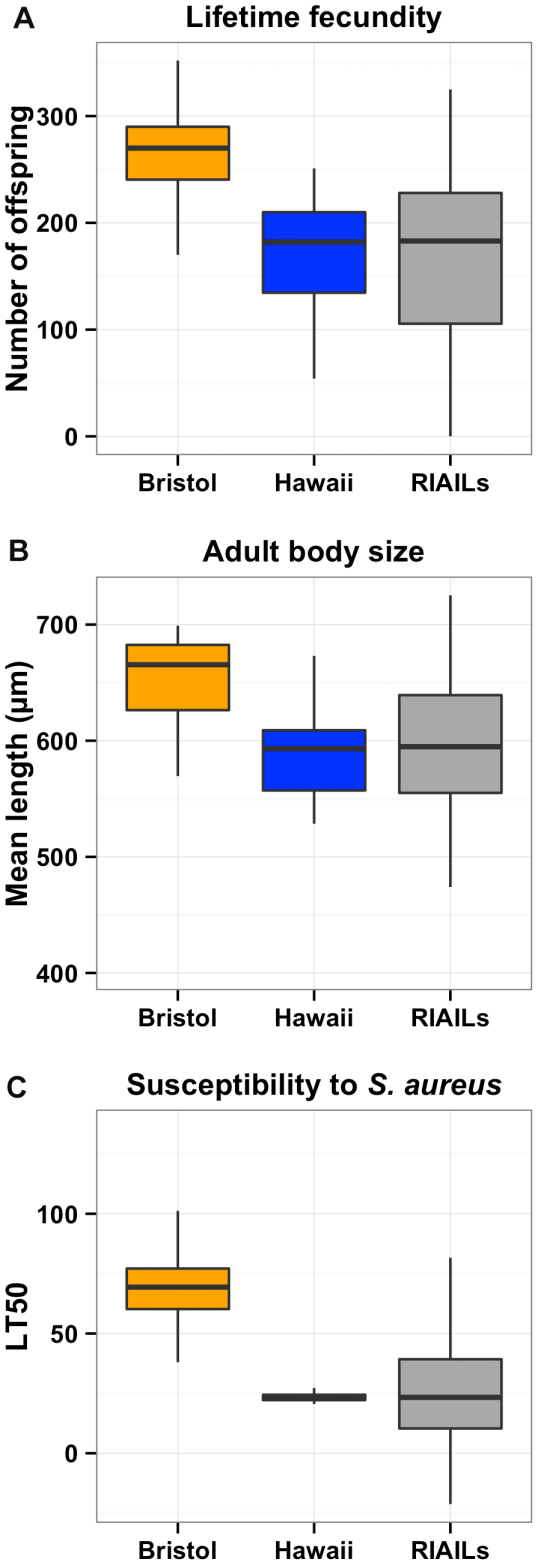
Phenotypic distributions of three quantitative traits for the Bristol and Hawaii parents along with the RIAILs. Box plots show the phenotypic distributions of the two parents (Bristol in orange and Hawaii in blue) and the recombinant inbred lines (RIAILs, dark gray) for lifetime fecundity (**A**), the length of adult animals (**B**), and the LT50 distribution after exposure to *S. aureus* (**C**). For each comparison between Bristol and Hawaii, the phenotypes are significantly different by Tukey's HSD with each *p*-value less than 2E-16.

The N2 strain from Bristol, England and the CB4856 strain from Hawaii, U.S.A. differ significantly in many traits [16]. We phenotyped these two strains for lifetime fecundity and adult body size. The Bristol strain had more offspring and grew larger than the Hawaii strain when grown in standard laboratory conditions (Figures 1A and B). Additionally, we found that the growth difference between the Bristol and Hawaii strains is present from the first larval stage through adulthood (Figure S1). We previously identified a difference in susceptibilities to the opportunistic human pathogen *Pseudomonas aeruginosa* between the Bristol and Hawaii strains [8]. We extended this study to measure susceptibility to another human pathogen, the gram positive bacterium *Staphylococcus aureus*. Notably, the Bristol strain survived longer than the Hawaii strain when exposed to *S. aureus*, much like we had observed with *P. aeruginosa* (Figures 1C and S2). These phenotypic differences between the Bristol and Hawaii parents gave us the opportunity to identify the genetic variants that alter lifetime fecundity, adult body size, and resistance to *S. aureus*.

### Linkage mapping defines a single QTL controlling diverse phenotypic traits

**Figure 2:**
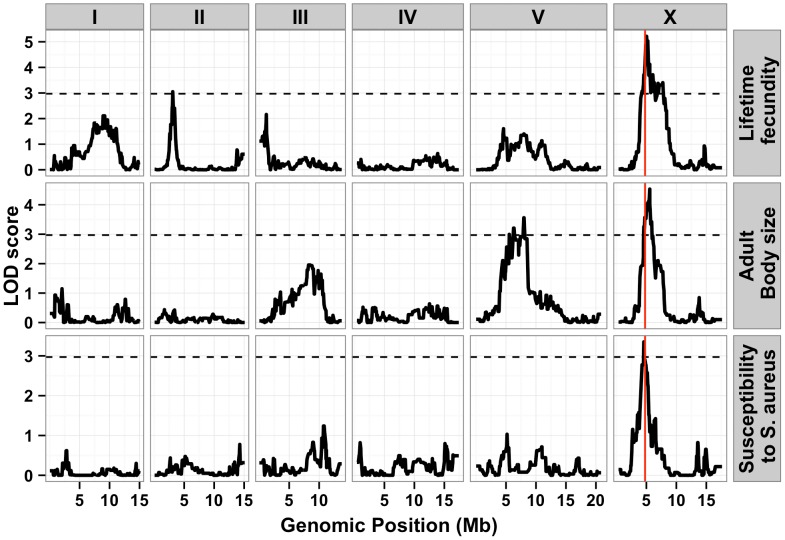
Linkage mapping results for lifetime fecundity, adult body size, and susceptibility to *S. aureus.* Linkage mapping results of the three traits are shown with genomic position (Mb) on the x-axis and LOD score on the y-axis. Each chromosome is in its own box labeled on top of the box. From top to bottom, the traits mapped are the lifetime fecundity, the mean length of adult animals, and the LT50 after exposure to *S. aureus*. The number of recombinant inbred lines (RIAILs) phenotyped are shown on the left of each plot. The dotted lines are the significance thresholds (genome-wide false positive rate of 0.05). The vertical red line is the position of the gene *npr-1*.

To identify these genetic variants, we scored a collection of advanced intercross recombinant inbred lines (RIAILs) that are mixtures of the Bristol and Hawaii genomes [15]. For all three traits, the phenotypic distributions of the RIAILs were closer to the mean parent phenotype of the Hawaii strain (Figure 1). Broad-sense heritability was 52% for adult fecundity, 57% for adult body size, and 72% for *S. aureus* sensitivity (see Methods). Next, we used linkage analysis to identify quantitative trait loci (QTL) that underlie the observed phenotypic differences. One or two significant QTL were detected for each trait (Figure 2). Remarkably, each of the three traits was influenced by a QTL in a shared region on chromosome X (QTL_X_). The confidence intervals for the location of these QTL_X_ overlapped substantially (Table 1). Lifetime fecundity and adult body size also mapped to other QTL, respectively on chromosomes II and V. No other significant QTL were detected (see Methods). Statistical power calculations using 150 RIAILs show that 80% of QTL that account for 20% of phenotypic variance should have been detected. The detected QTL explained 18% to 33% of broad-sense heritability (adult fecundity: QTL_X_ = 28%, QTL_II_ = 18%; adult body size QTL_X_ = 23%, QTL_V_ = 23%; *S. aureus* sensitivity: QTL_X_ = 33%). Therefore, the detected QTL likely represent most, if not all, of the large-effect QTL controlling these quantitative traits.

**Table 1:** Attributes for all significant quantitative trait loci are described.

Trait	Broad-sense heritability (SE)	QTL	Chromosome	Variance explained	Confidence interval	
					Left marker	Right marker
Lifetime fecundity	52%±12%	1	X	28%	4571085	7743234
Lifetime fecundity	52%±12%	2	II	18%	2601221	3576981
Adult body size	57%±23%	1	X	23%	4571085	5877800
Adult body size	57%±23%	2	V	23%	4296831	8498661
Susceptibility to *S. aureus*	71%±11%	1	X	33%	4753766	5414458

### *npr-1* is the causal gene underlying differences in the three traits

**Figure 3:**
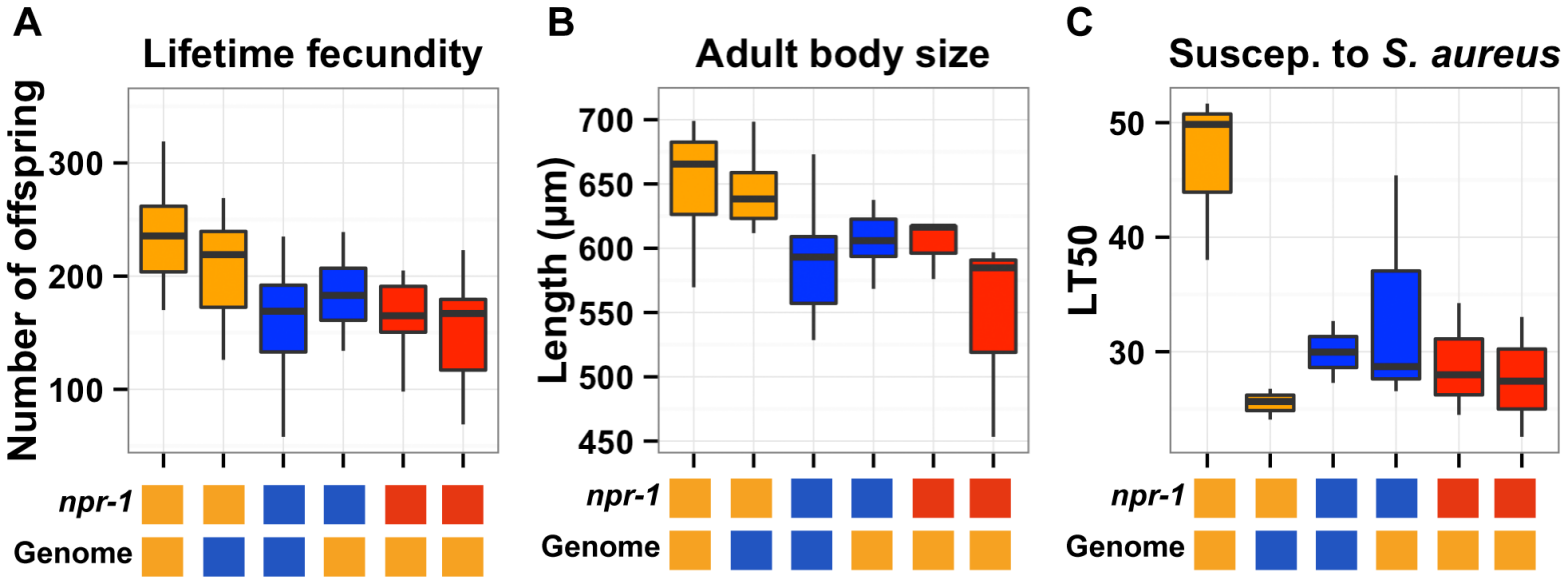
Phenotypic distributions of three quantitative traits for the parents, nearly isogenic lines, and *npr-1* mutants. Box plots show the summary phenotype data of the two parents (Bristol in orange and Hawaii in blue), the two nearly isogenic lines (*kyIR9* in orange and *qgIR1* in blue), and the two independent *npr-1 loss-of-function alleles (npr-1(ad609) and npr-1(ky13) in red). From left to right, the lifetime fecundity (**A**), the mean length of adult animals (**B**), and the LT50 distribution after exposure to S. aureus (**C**). Below each plot are two boxes. The top box denotes the npr-1 genotype: laboratory-derived NPR-1V from Bristol in orange, ancestral wild-type NPR-1F from Hawaii in blue, or loss-of-function allele from Bristol in red. The bottom box denotes the genome-wide genotype: Bristol in orange and Hawaii in blue. Statistical significance was tested using Tukey's HSD. For (**A**), the qgIR1 strain has significantly fewer offspring than the Bristol strain (p = 0.003) does, and the kyIR9 strain has significantly more offspring than the Hawaii strain (p = 0.0059) does. The Hawaii strain, qgIR1, and the two npr-1 loss-of-function alleles do not have significantly different numbers of offspring. The same is true for the Bristol strain and kyIR9. For (**B**), the qgIR1 strain is significantly smaller than the Bristol strain (p = 0.00148), and the kyIR9 strain is significantly larger than the Hawaii strain (p = 6E-5). The Hawaii strain, qgIR1, and the two npr-1 loss-of-function alleles do not have significantly different lengths. The same is true for the Bristol strain and kyIR9 introgression strain.*

The overlapping confidence intervals of the QTL on the X chromosome contain the gene *npr-1*. Given that variation in *npr-1* is known to cause phenotypic differences in multiple other traits [5], [7], [9], [21]–[28], we sought to determine whether variation in *npr-1* also caused differences in the three traits described here. To narrow each QTL on the X chromosome, we took advantage of two nearly isogenic lines (NILs or congenics) in which the genome is derived from one strain background, except for a small region of the X chromosome that is derived from the other strain. The *qgIR1* NIL is Bristol throughout its genome and Hawaii for a portion of the QTL_X_ confidence interval. The *kyIR9* NIL is Hawaii throughout its genome and Bristol for a portion of the QTL_X_ confidence interval. We did two comparisons that test the *npr-1* interval for causality in each of the three traits: we compared the Bristol parent to *qgIR1* and the Hawaii parent to *kyIR9* (Figure 3).

For lifetime fecundity, the Bristol parent had a mean brood size of 236 offspring compared to 177 offspring in the *qgIR1* NIL that contains the Hawaii version of *npr-1*. This comparison shows that the Hawaii version of the introgressed region causes a 25% decrease in fecundity. The Hawaii parent had a mean brood size of 160 offspring, compared to 206 offspring in the *kyIR9* NIL that contains the Bristol version of *npr-1*. This comparison shows that the Bristol version of the introgressed region causes a 29% increase in fecundity. Both of these comparisons were significant using Tukey's HSD test, with *p* = 0.0003 and *p* = 0.0059, respectively. The RIAILs with the Bristol allele at the most significantly linked marker had a mean brood size of 195 offspring, compared to 146 offspring for the RIAILs with the Hawaii allele, indicating that in the linkage mapping experiment the Hawaii allele caused a 25% decrease in fecundity. Thus, the fecundity differences between the NILs closely matched the phenotypic effect observed in the RIAILs.

For adult body size, the Bristol parent had a mean body length of 654 µm, compared to 604 µm in the *qgIR1* NIL. This comparison shows that the Hawaii version of the introgressed region causes an 8% decrease in adult body size. The Hawaii parent had a mean body length of 587 µm, compared to 646 µm in the *kyIR9* NIL. This comparison shows that the Bristol version of the introgressed region causes a 10% increase in adult body length. Both of these comparisons were significant using Tukey's HSD test, with *p* = 0.00148 and *p* = 6E-5, respectively. The RIAILs with the Bristol allele at the most significantly linked marker had a mean body length of 619 µm, compared to 578 µm for the RIAILs with the Hawaii allele, indicating that in the linkage mapping experiment the Hawaii allele caused a 7% decrease in mean body length. Just as with lifetime fecundity, the mean body length differences between the NILs closely matched the phenotypic effect observed in the RIAILs.

For susceptibility to *S. aureus*, the Bristol parent had a mean LT50 of 47 hours, compared to 34 hours in the *qgIR1* NIL. This comparison shows that the Hawaii version of the introgressed region causes a 28% reduction in survival after exposure to *S. aureus*. The Hawaii parent had a mean LT50 of 30 hours, compared to 26 hours in the *kyIR9* NIL. Unexpectedly, this comparison shows that the Bristol version of the introgressed region causes a 13% reduction in survival after exposure to *S. aureus*. We expected that the *kyIR9* NIL with the QTL region derived from the Bristol strain would be more resistant than the Hawaii strain. We believe that we observed this difference for two reasons: (1) the *kyIR9* NIL is slower and more uncoordinated than the *qgIR1* NIL (R. Ghosh and L. Kruglyak, unpublished results) and spends more time on the deadly pathogen lawn, and (2) Hawaii alleles at other loci may interact with Bristol alleles of genes in this NIL region to make the animals more susceptible to *S. aureus*. The RIAILs with the Bristol allele at the most significantly linked marker had a mean LT50 of 41 hours, compared to 18 hours for the RIAILs with the Hawaii allele, indicating that in the linkage mapping experiment the Hawaii allele caused a 56% decrease in the mean LT50. This effect was much larger than observed using the NILs, consistent with a putative genetic interaction between this locus and the Hawaii background.

The NIL results indicate that the smallest region covered by the two NILs likely contains the gene with a causal role in the three phenotypic differences. The *qgIR1* NIL has the smallest introgressed region, with seven genes from the Hawaii strain in an otherwise Bristol genetic background. Of these seven genes, only *npr-1* and *R08E3.3* have non-synonymous changes (comparison to WS210 sequence), and only *R08E3.3* has a local gene expression QTL [1]. We focused our analyses on these two genes. RNAi knockdown or deletion of *R08E3.3* does not affect fecundity, body size, and movement, nor cause any other obvious phenotypic effect [29], [30]. For this reason, we tested the role of *npr-1* in lifetime fecundity, adult body size, and susceptibility to *S. aureus*.

To determine whether variation in the gene *npr-1* causes these phenotypic differences, we used two independent loss-of-function alleles of *npr-1*, *ad609* and *ky13*, derived in the Bristol genetic background. These two putative null alleles remove the dominant novel activity of *npr-1* from the Bristol strain, and each strain behaves like the Hawaii strain by aggregating in regions of low oxygen at the edge of bacterial lawn [21]. For each of the three traits, the *npr-1* loss-of-function alleles had similar phenotypes to the Hawaii parent, with effect sizes nearly matching those observed in the NILs (Figure 3). Therefore, variation in *npr-1* is the cause of these large phenotypic differences observed between the Bristol and Hawaii strains.

### *npr-1*-mediated aggregation causes drastic effects on the growth and physiology of *C. elegans*

The laboratory-derived allele of *npr-1* present in Bristol causes animals to disperse across the *E. coli* bacterial lawn, whereas wild *C. elegans* strains aggregate at the edges of the bacterial lawn. This aggregation behavior is caused by a preference for low oxygen found in natural strains of *C. elegans*, and the edge of the bacterial lawn has a low oxygen concentration of approximately 10% [24]. By contrast, the rest of the agar plate and bacterial lawn have an oxygen concentration of approximately 21% oxygen. When the Hawaii strain is reared at 10% oxygen, it behaves like the Bristol strain and spreads out across the bacterial lawn because there is no longer a difference in oxygen concentration across the plate environment [8]. Previously, we showed that this oxygen preference behavior mediates differences in survival after exposure to *P. aeruginosa* [8]. We hypothesized that this “isolation” behavior also mediates the trait differences observed in this study.

**Figure 4:**
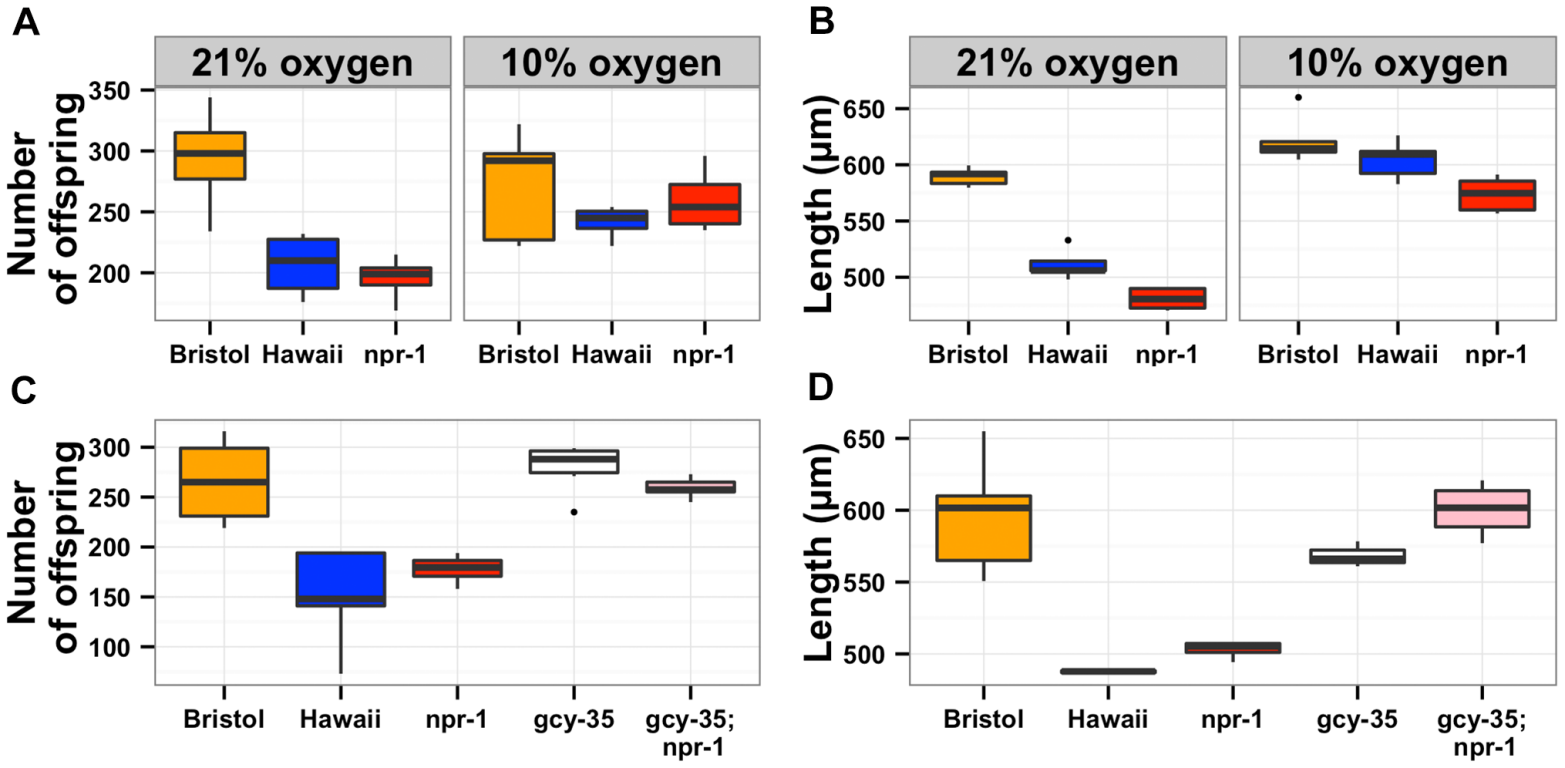
The effects of aerotaxis behaviors on lifetime fecundity and adult body size. The oxygen avoidance behavior mediated by *npr-1* is necessary for alterations in lifetime fecundity (**A**) and adult body size (**B**). The box plots show summary data from assays performed at 21% ambient oxygen (left) versus 10% oxygen (right). At 10% oxygen, the Hawaii strain no longer clumps and borders instead spreading out across the entire surface of the agar plate while consuming bacterial food. At this lower oxygen level, the Hawaii strain and a *npr-1* loss-of-function allele do not have statistically different lifetime fecundities (*p* = 0.37 and *p* = 0.07) from the Bristol strain. Adult body size is not significantly different between Bristol and Hawaii (*p* = 0.31) but is different between Bristol and *npr-1(ad609)* (*p* = 0.003). However, the large increase in adult body size by oxygen concentration suggests that clumping largely influences this trait. A mutation that causes failure to sense oxygen, *gcy-35(ok769)*, suppresses the lifetime fecundity (**C**) and adult body size (**D**) defects of *npr-1(ad609)*. The double mutant *gcy-35(ok769); npr-1(ad609)* has significantly more offspring than the *npr-1(ad609)* single mutant (*p* = 0.005) and grows significantly larger than the *npr-1(ad609)* single mutant (*p* = 0.0001). All statistical significance was assessed by Tukey's HSD test.

In order to determine whether the oxygen preference behavior also controlled lifetime fecundity, adult body size, and susceptibility to *S. aureus*, we needed to reliably assay these traits at both 21% and 10% oxygen concentrations. The oxygen chamber had different humidity and temperature conditions than the standard *S. aureus* assay conditions, which altered the experimental results. Thus, we did not assay susceptibility to *S. aureus* at low oxygen concentration. For lifetime fecundity and adult body size assays, we raised the Bristol and Hawaii parent strains along with the *npr-1* loss-of-function strain *npr-1(ad609)* in either ambient (21%) or low (10%) oxygen while consuming *E. coli* bacteria (see Methods). The reductions in lifetime fecundity observed in the Hawaii and *npr-1(ad609)* strains as compared to the Bristol strain were completely abrogated by rearing the animals at 10% oxygen (Figure 4A). Shifting the oxygen concentration was sufficient to increase the Hawaii brood size by 20% and the *npr-1(ad609)* brood size by 32%. The effect of *npr-1* on this trait was thus eliminated by lowering the oxygen concentration and making animals disperse across the bacterial lawn. For adult body size, animals reared at 10% oxygen were significantly longer, and the reductions in mean body length observed in the Hawaii and *npr-1(ad609)* strains as compared to the Bristol strain were completely eliminated (Figure 4B). The Hawaii and *npr-1(ad609)* strains increase mean body length 18% and 19%, respectively, when compared to growth at 21% oxygen. To further test whether these differences in fecundity and body size are mediated by oxygen concentration differences, we tested whether loss of the oxygen-sensing gene *gcy-35*, which can suppress the aggregation behavior of *npr-1(ad609)* mutants [31], [32], can suppress the fecundity and body size defects as well. We observed that *gcy-35(ok769); npr-1(ad609)* double mutants had significantly more offspring (*p* = 0.005) and grew significantly larger (*p* = 0.0001) than *npr-1(ad609)* single mutants (Figures 4C and D).

**Figure 5:**
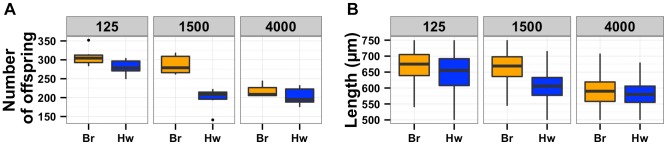
Population density directly impacts lifetime fecundity and adult body size. Box plots for Bristol (orange) and Hawaii (blue) strains are shown for low, normal, and high densities of 125, 1500, or 4000 animals per 10 cm agar plate, respectively. (**A**) Decreased culture density caused a significant increase in fecundity (*p* = 1.3E-4) of the Hawaii strain from 198 to 280 offspring. By contrast, increased culture density did not cause a significant difference (*p* = 0.95) in fecundity of the Hawaii strain. (**B**) Decreased culture density (125 vs. 1500 animals) caused a significant increase in adult body size (*p* = 2E-16) of the Hawaii strain from 606 µm to 649 µm. By contrast, increased culture density (4000 vs. 1500 animals) caused a significant decrease (*p* = 2E-16) in adult body size of the Hawaii strain from 606 µm to 583 µm.

The results of the oxygen experiments described above suggest that aggregation causes significant reductions in growth and offspring production. Aggregation is dependent on the density of animals grown on agar plate cultures [21], [22]. At low culture density of 125 animals per 10 cm agar plate, the Hawaii strain does not aggregate. By contrast, at high culture density of 400 animals per 10 cm agar plate, that strain readily aggregates at the edge of the bacterial lawn. To test whether differences in lifetime fecundity and adult body size between the Bristol and Hawaii strains depend on aggregation, we cultured animals at low (125), normal (1500), and high (4000) density (Figure 5). At low density, the Hawaii strain has more offspring and grows 7% larger than when reared at normal density. At high density, the Hawaii strain has approximately the same number of offspring and grows only 3% smaller than when grown at normal density. These results suggest that strains consuming standard *E. coli* bacterial food in large aggregates grow smaller and are less fecund than strains that do not grow in such aggregates.

### Aggregation likely causes chronic underfeeding, which affects expression of signaling pathways and animal growth

**Figure 6:**
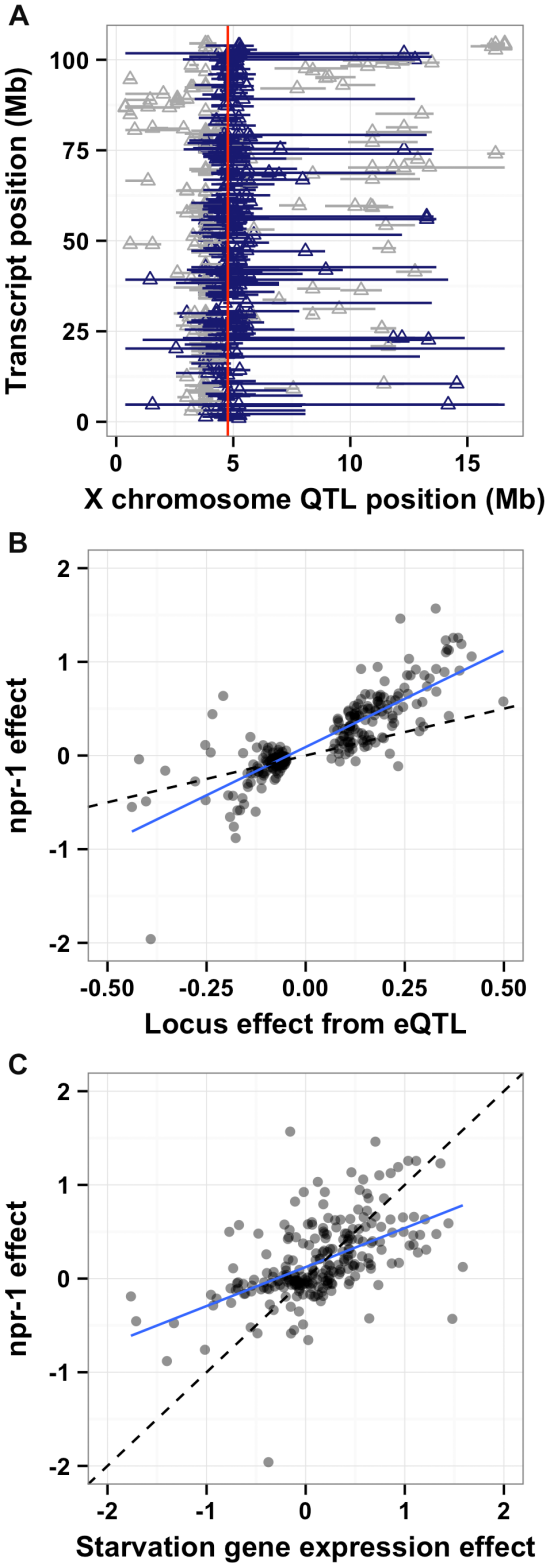
*npr-1* underlies many expression QTL and could cause chronic low-level starvation. (A) The physical positions of gene expression QTL 95% confidence intervals on the X chromosome (Mb) on the x-axis are plotted against the physical genomic location of the transcript (Mb) on the y-axis. Triangles indicate the position of the maximum LOD score for that QTL. Blue horizontal bars denote confidence intervals that overlap with the physical position of *npr-1* (shown as a red vertical line). Gray horizontal bars denote confidence intervals that do not overlap with the physical position of *npr-1*. (**B**) Scatterplot of the locus effect from the eQTL mapping versus the effect of *npr-1* for the 247 genes with eQTL confidence intervals overlapping *npr-1*. Each point represents a transcript. The blue line is the best fit by linear regression. The dotted black line indicates y = x. The slope of the regression line is 1.82 (95% CI 1.72–1.91) with a correlation of 0.83 (59% variance explained). (**C**) Scatterplot of the effect of starvation versus the effect of *npr-1* for the 247 genes with eQTL confidence intervals overlapping *npr-1*. Symbols and lines as in (B). The slope of the regression line is 0.76 (95% CI 0.68–0.84) with a correlation of 0.61 (28% variance explained).

One promising approach to connect DNA sequence variation to phenotypic variation is analysis of gene expression QTL (eQTL) [33]. Expression traits can implicate a known pathway in the physiological process that is altered by the variant and provide insight into how the variant causes phenotypic differences. Previously, we mapped thousands of eQTL between the Bristol and Hawaii strains [1]. A reanalysis of these data (see Methods) detected significant eQTL (false discovery rate = 5%) for 247 gene expression traits with confidence intervals that overlapped *npr-1* (Figure 6A and Table S1). To determine whether differences in the function of *npr-1* cause these gene expression differences, we profiled gene expression in the Bristol parent strain, the *qgIR1* NIL (with the Hawaii version of *npr-1* in a Bristol genetic background), and the predicted *npr-1* loss-of-function allele *ad609*. The gene expression differences between the Bristol strain and the NIL or the Bristol strain and the predicted null allele should largely depend on *npr-1*. We estimated the *npr-1* effects by fitting a linear model to the gene expression traits of these three strains (see Methods). Then, we compared the effect of the altered *npr-1* function to the effect observed in the RIAILs (Figure 6B). We observed high correlations between the gene expression effects from altered *npr-1* function (observed in the NIL or the loss-of-function allele) and the RIAIL effects for transcripts linked to the *npr-1* locus. The regression slope was 1.82 (rho = 0.83), indicating a strong positive relationship. Thus, polymorphism in *npr-1* is the major contributor to the gene expression differences observed for these 247 genes. These genes are significantly enriched for several gene ontology (GO) classes: neuropeptide and insulin signaling, transferring hexosyl groups (central carbon metabolism), and metabolic process (Table S1), suggesting that growth rate is one of the major physiological effects of altered *npr-1* function and aggregation behaviors.

**Figure 7:**
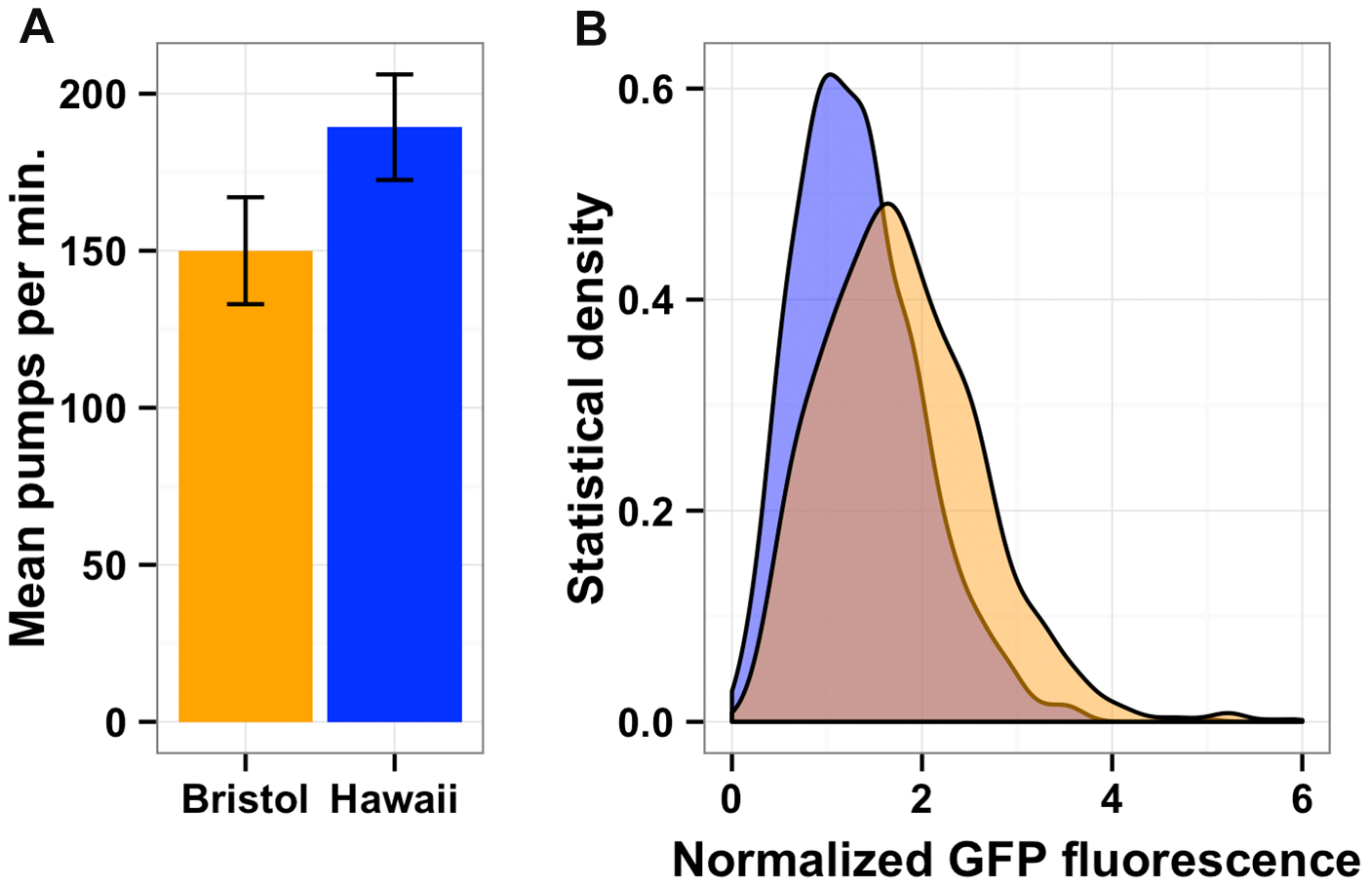
Bacterial food consumption differs between the Bristol and Hawaii strains. **(A)** Average pharynx pumps per minute for Bristol (orange) and Hawaii (blue) are shown and are significantly different by Tukey's HSD test (*p* = 2E-16). (**B**) Both the Bristol and Hawaii strain were fed *E. coli* bacteria expressing GFP (HB101-GFP) for two hours. The statistical density of green fluorescence normalized by length is shown for Hawaii (blue) and Bristol (orange).

Reduced food availability or starvation is sufficient to decrease growth rates and fecundity in a variety of organisms, including *C. elegans* [34]. We hypothesized that the Hawaii strain might be chronically underfed, because in large aggregates bacterial food is depleted locally. To test this hypothesis, we obtained data on gene expression differences observed between starved and well fed Bristol animals [35] for the 247 genes whose expression linked to *npr-1*. Then, we compared the effect of the altered *npr-1* function to the effect observed in the starvation experiments (Figure 6C). We observed positive correlations between the gene expression effects from altered *npr-1* function and the effects of starvation on the Bristol strain. The regression slope was 0.76 (rho = 0.61), indicating a strong positive relationship and suggesting that at least some of the gene expression differences observed between strains with the laboratory-version of *npr-1* and strains with the natural allele of *npr-1* could be explained by reduced food availability. Starvation is known to alter pharyngeal pumping rate [36], [37] with starved animals pumping more once bacterial food is encountered again. We observed that the Hawaii strain pumps significantly more (*p* = 2E-16) than than Bristol strain (Figure 7A). This difference suggests that the Hawaii strain is underfed. To test how much the Hawaii strain eats when aggregated, we fed both the Bristol and Hawaii strains an *E. coli* bacterial food source expressing GFP. Using the COPAS Biosort, we measured the amount of green fluorescence per unit length inside each animal in the culture. Even though the Hawaii strain pumps more, the strain consumes less food than the Bristol strain (Figure 7B), likely caused by increased competition for food inside aggregates.

## Discussion

We used linkage mapping to identify QTL that control differences in lifetime fecundity, adult body size, susceptibility to the human pathogen *S. aureus*, as well as expression of hundreds of genes between the Bristol laboratory strain (N2) and a Hawaii wild strain (CB4856). Taking advantage of reciprocal NILs and two loss-of-function alleles, we identified the neuropeptide receptor gene *npr-1* as the quantitative trait gene underlying a QTL shared by all of these traits. Additionally, we showed that these large phenotypic effects are caused by variation in aggregation and feeding behaviors found between the Bristol and Hawaii strains mediated by *npr-1*.

The laboratory-derived variant of NPR-1 found in the Bristol strain is abnormally sensitive to an additional neuropeptide [20], creating a constitutively activated neural circuit through the RMG interneuron [22]. This hyperactive circuit causes Bristol animals to avoid each other and the edges of the bacterial lawn when consuming food. By contrast, the Hawaii strain aggregates at the edge of the bacterial lawn. The increased density of animals in an aggregate at the edge of the bacterial lawn may cause animals to deplete food faster than if they were spread across the bacterial lawn. Such local food depletion can cause physiological responses to decreased food [34], [38], [39], which can decrease fecundity and growth rate. We showed that the Hawaii strain consumes less food even though the strain has high pharyngeal pumping activity than the Bristol strain. These results suggest that the natural strains of *C. elegans* including the Hawaii strain (CB4856) are chronically underfed when reared in standard laboratory conditions. In support of this hypothesis, we observed a significant correlation between gene expression changes during starvation and those observed in strains with the Hawaii version of *npr-1* on an abundant food source. Moreover, the genes that change expression as a result of *npr-1* variation are enriched for neuropeptide signaling and growth. It is likely that animals in large aggregates locally deplete bacterial food at a faster rate than solitary animals, enter a physiological state similar to weak starvation, and decrease offspring production and growth rates to adapt to the decreased availability of food. Once the animals encounter a larger supply of food, growth and offspring production resume. Alternatively, it is possible that animals in large aggregates have reduced growth and offspring production because they experience higher local pheromone levels, indicating more competition from peers. We must perturb the putative pheromone receptor that controls growth and offspring production to make this functional connect. Thus far, we can not differentiate these alternative hypotheses. Regardless, the laboratory variant of *npr-1* has large and diverse pleiotropic effects on the growth and physiology of the animal because it fundamentally alters behavior. These results illustrate how behavioral changes can translate to broad physiological effects, and provide a case study of the molecular and physiological mechanisms that underlie pleiotropy.

The highly pleiotropic laboratory allele of *npr-1* affects growth and physiology of the Bristol strain long used by most *C. elegans* research laboratories. Given these large effects, it is not difficult to hypothesize how this particular allele was selected in the laboratory. Compared to wild *C. elegans* strains, the dispersal behavior of the Bristol laboratory strain makes it easier to select solitary individuals for maintenance or genetic crosses [7]. Others found that this strain is well adapted in a continuous food environment found in a research setting [40]. Additionally, we found that the Bristol strain has an increase in lifetime fecundity and enhanced survival after exposure to pathogens when grown under laboratory conditions. Lab-derived alleles of large-effect have also been observed in the standard laboratory strains of other common model organisms. For instance, in *S. cerevisiae*, loss-of-function mutations in *AMN1* [17] and *FLO8* [18] reduce two aggregation phenotypes—clumping and flocculation—and make S288C and related strains much easier to use in a laboratory setting. Similarly, the commonly used *A. thaliana* accession, Landsberg erecta, has EMS-derived mutations in the gene *ERECTA* [41], which enables the plants to grow more robustly in laboratory incubators [19]. In both of these organisms, just like in *C. elegans*, mapping studies employing recombinant inbred line panels that include these laboratory strains often find these derived alleles as major-effect QTL.

The Bristol strain has other laboratory-derived alleles with large phenotypic effects on the animal, including variants that alter oxygen and carbon dioxide avoidance [7] and fecundity [10], as well as many predicted functional variants [42], [43]. The large effects of these alleles in crosses using laboratory-derived strains could obscure the more modest phenotypic effects of natural alleles [44] and hinder their detection. Therefore, studies aimed at understanding natural genetic variation in *C. elegans* need to complement recombinant inbred line panels involving the Bristol strain with new panels derived from crosses using wild isolates. Additionally, it is important to devise assays of physiological traits that are not confounded by the effects of feeding behavior as observed on agar plates with a lawn of ample bacterial food. Because the standard Bristol strain has an abnormally active RMG neural circuit caused by high NPR-1 activity, behaviors observed in this strain may be idiosyncratic and not shared by wild strains, making the results of many behavioral studies harder to interpret in an evolutionary context.

## Materials and Methods

### Strains

Animals were cultured at 20°C with the bacterial strain OP50 on modified nematode growth medium (NGM), containing 1% agar and 0.7% agarose to prevent burrowing of the Hawaii strain and *npr-1* loss-of-function strains. For each assay, strains were grown at ambient atmospheric conditions (*i.e.* 21% oxygen) for at least five generations with no strain entering starvation or encountering dauer-inducing conditions. To investigate the causality of *npr-1* in our traits, we used two putative null alleles of *npr-1*: *ky13*, which has a C-to-T transition that introduces a stop codon after the first transmembrane domain; and *ad609*, which has two missense mutations - a threonine-to-isoleucine change in transmembrane domain two and a threonine-to-alanine change in transmembrane domain four [21].

### Quantitative phenotyping

#### Lifetime fecundity assays

A modified NGM recipe with 2% agarose substituted for agar was used for all fecundity assays. Without this substitution, the Hawaii strain, RIAILs with the Hawaii version of *npr-1*, and *npr-1* loss-of-function strains burrow readily and fecundity is difficult to measure accurately. 100 µL of overnight OP50 culture was spotted on each plate and dried in a laminar flow hood. For each genotype, single L4 larval stage hermaphrodites were picked to each of six plates. Plates were kept at 20°C for the duration of the assay. For each assay plate, the original adult hermaphrodite parent was picked to a fresh plate after 48 and 96 hours. The total offspring from each of these three plates was counted manually. Most strains had very few offspring after 96 hours. For population density experiments, assays were prepared and measured as described above, except animals were picked from 10 cm plates where population densities were defined to be 125, 1500, or 4000 animals per plate.

#### Adult body size assays

25–30 L4 hermaphrodites from each strain were picked to both of two 10 cm modified NGM agar plates. Strains were grown for four to five days and bleached to collect a approximately synchronous embryo population. Embryos were hatched for 15–24 hours at 20°C on a roller drum at a density of one embryo/µL in a total of 2 mL S medium. The hatched and arrested L1 larvae were plated onto three 10 cm plates at a density of 1500 animals/plate. Strains were grown for 72 hours at 20°C. Most of the animals were day-one adults when length and optical density was assessed using a COPAS Biosort (Union Biometrica). All objects smaller than 100 µm or 100 extinction units were removed from the analysis. Most of these objects were next generation L1 animals. Custom R scripts processed the data to obtain mean and median length and optical density values. Because the mean length was more heritable than any other measure, we used mean length for all future size calculations. Population density experiments were prepared and measured as described above, except a defined number of L1 larvae (125, 1500, or 4000) were plated per 10 cm modified NGM agar plate. The plates with 4000 animals were checked regularly and moved to fresh 10 cm plates if food became limiting.

#### *Staphylococcus aureus* survival assays

*Staphylococcus aureus* assay plates were made of brain heart infusion (BHI) agar (Gibco #211065) supplemented with Bactoagar (Gibco #214010) to a final agar concentration of 1.7%. The autoclaved agar was supplemented with nalidixic acid to a final concentration of 10 µg/mL and poured into 3.5 cm petri plates. *S. aureus* strain NCTC8325 was grown for 24 hours in BHI liquid media with nalidixic acid. Subsequently, 10 µL of bacterial suspension was spread onto the surface of the assay plates and grown at 37°C for 24 hours. After growth at 37°C, assay plates were kept at room temperature for no more than 24 hours at which point 30 L4 stage animals were added to each assay plate. The plates were incubated at 25°C and the fraction dead was scored every 12 hours from 0 to 48 hours. The LT50 was determined by fitting an exponential decay formula used previously [8].

#### Pharyngeal pumping assays

The pharynges of ten adult animals (24 hours after the fourth larval stage) were observed for one minute each in three separate trials. Animals were reared at 1500–2000 animals per 10 cm plate before each assay. Data were tabulated and analyzed using R.

#### Feeding rates

Animals were reared at high density (as before) on plates seeded with OP50. After washing off those plates, animals were plated onto our modified NGM plates seeded with HB101 expressing GFP at 2000 animals per plate. At this density, animals clumped within five minutes of plating. Two hours after plating out day-one adults, animals were washed off and assayed for green fluorescence using the COPAS Biosort. Green fluorescence was normalized by animal length (TOF parameter).

### Quantitative trait mapping

Sets of advanced intercross recombinant inbred lines (RIAILs) from a cross between the Bristol (N2) and Hawaii (CB4856) strain were phenotyped as described above. The phenotype data and RIAIL genotype data [15] were entered into the R/qtl package [45]. In most cases, the phenotypes of the RIAILs were approximately normally distributed. QTL were detected using interval mapping [46]. The 5% genome-wide significance threshold was calculated based on 500 permutations of the phenotype data [47]. The marker with the highest LOD score was used as a covariate to identify additional QTL until no more significant QTL were detected. QTL model fitting and covariate analysis used normal models with the Haley-Knott estimator [48]. The fits were used to estimate the total phenotypic variance explained by each QTL. Broad-sense heritability was calculated as the fraction of phenotypic variance explained by strain from fit of a linear mixed-model of repeat phenotypic measures of the parents and RIAILs [49]. The total variance explained by each QTL was divided by the broad-sense heritability to determine how much of the heritability is explained by each QTL. Confidence intervals were defined as the regions contained within a 1.5 LOD drop from the maximum LOD score. 73, 145, and 109 RIAILs were scored for lifetime fecundity, adult body size, and susceptibility to *S. aureus*, respectively.

### *npr-1* causality experiments

Causality experiments compared phenotypes of six strains: the two parents (Bristol and Hawaii), the two NILs (*qgIR1* and *kyIR9*), and the two loss-of-function alleles (*npr-1(ad609)* and *npr-1(ky13)*). The significance from every pairwise comparison was calculated using Tukey's Honest Significant Difference (HSD) test.

### Gene expression assays and quantitative trait mapping

Expression data were corrected for dye effects using a linear model and phenotypic residuals were used for downstream analysis. Probes with polymorphisms were removed. We tested for linkage by calculating LOD scores for each genotypic marker and each trait as -n(ln(1-R∧2)/2ln(10)) where R is the Pearson correlation coefficient between the RIAIL genotypes at the marker and RIAIL trait values, as calculated in [49]. To estimate significance empirically, assignment of phenotype to each RIAIL was randomly permuted 1000 times while maintaining the correlation structure among phenotypes [50]. The maximum LOD score for each chromosome and trait was retained. The FDR was calculated as the ratio of expected peaks to observed peaks across different LOD thresholds. Genetic markers corresponding to QTL peaks which were significant at an FDR of 5% were added to a linear model for each trait. Trait-specific linear models that included the significant QTL genotypes as additive covariates were computed, and phenotypic residuals were estimated. Phenotypic residuals for each trait were then used for another round of QTL detection. This process of peak detection, calculation of empirical significance thresholds, and expansion of the linear model for each trait to include significant QTLs detected at each step was repeated three times. The LOD thresholds corresponding to a 5% FDR at each step were 3.8, 4.98, and 7.5. 2,447 QTL were detected by these analyses. Approximate 95% confidence intervals were determined using a drop of one LOD value. The confidence intervals of 247 QTL overlapped with the position of *npr-1* and have genomic positions further than 10 kb from the QTL peak (Table S1).

RNA was collected and microarrays performed as described previously [1] for the strains Bristol, Hawaii, *qgIR1*, *kyIR9*, *npr-1(ad609)*, and *npr-1(ky13)*. Microarrays were processed using the R package Limma [51]. Non-uniform outliers and probes with signal well below background were removed. Median signals were background corrected. Within-array normalization was performed using the “loess” method. Between-array normalization was performed using the “Gquantile” method. Duplicate probes were averaged. Probes with SNPs between the Bristol and Hawaii strains were removed from the analysis. All microarray data are available through GEO (GSE49307).

For each of the 247 QTL identified previously, a linear model was fit per gene accounting for strain background and *npr-1* status (Bristol-like or Hawaii-like) as covariates. Loss-of-function strains were considered to have a Bristol genetic background and Hawaii-like *npr-1* status. Effect sizes were calculated as the multiple regression coefficient for *npr-1* status. The Spearman correlation was calculated comparing these regression coefficients to the differences in expression from the eQTL results.

For the comparison to starvation gene expression, we used the GEOquery R package to download Agilent microarray data from GSE15656 [35] and extracted the gene expression ratios of six-hour starved to well fed L4 larvae. These ratios were compared to the effect sizes of the genes with distant QTL overlapping *npr-1*. Because we compared a starvation experiment using L4 larvae to our experiment using adult worms, we also analyzed the correlation between gene expression ratios from well fed L4 larvae [52] and our effect sizes. This correlation was much lower (rho = 0.22).

### Gene ontology class enrichment

Using ProfCom [53], gene expression traits were analyzed for enrichment of GO terms. The following GO terms and reference numbers were significantly enriched: neuropeptide signaling pathway (0007218), transferring hexosyl groups (0016758), and metabolic process (0008152). The respective *p*-values were 5.47e-12, 2.89e-2, and 3.32e-2.

### Oxygen concentration assays

Adult body size and lifetime fecundity were assessed as described above, except paired assays were prepared at 10% and 21% oxygen using a Coy hypoxia chamber with oxygen analyzer (Coy Laboratory Products, Grass Lake, MI). Oxygen was kept constant using a nitrogen gas source. The temperature inside the oxygen chamber was approximately 23.5°C, as opposed to 20°C in the incubator. For this reason, strains grow faster inside the oxygen chamber and paired comparisons for body size and fecundity to 21% oxygen were not appropriate. All statistical comparisons were performed within the oxygen growth conditions and not between. Given the differences in temperature and humidity between ambient and 10% oxygen, we did not assay susceptibility to *S. aureus* where assays are normally performed at 25°C.

## Supporting Information

Figure S1 We observed that the Bristol strain (orange) is larger than the Hawaii strain (blue) at all-time points throughout *C. elegans* development, except for the zero hour arrested L1 time point. Size differences of L1 larvae are likely determined largely by maternal effects. In three independent assays, we observed examples of both the Bristol strain being smaller and larger than the Hawaii strain. For this reason, we indicated the uncertainty with an asterisk. The *p*-value for each comparison (Tukey's HSD) is shown above the boxplots for each time point.
(PDF)

Figure S2 An example survival curve of Bristol (orange) and Hawaii (blue) after exposure to the opportunistic human pathogen *S. aureus*. The dotted line denotes when half of the population was alive or dead.
(PDF)

Table S1 Data for all 247 gene expression traits are listed.
(XLS)

## References

[1] Rockman MV, Skrovanek SS, Kruglyak L (2010) Selection at linked sites shapes heritable phenotypic variation in C. elegans. Science 330: 372–376 doi:10.1126/science.1194208 20947766 10.1126/science.1194208 PMC3138179

[2] Seidel HS, Ailion M, Li J, van Oudenaarden A, Rockman MV, et al (2011) A novel sperm-delivered toxin causes late-stage embryo lethality and transmission ratio distortion in C. elegans. Plos Biol 9: e1001115 doi:10.1371/journal.pbio.1001115 21814493 10.1371/journal.pbio.1001115 PMC3144186

[3] Seidel HS, Rockman MV, Kruglyak L (2008) Widespread genetic incompatibility in C. elegans maintained by balancing selection. Science 319: 589–594 doi:10.1126/science.1151107 18187622 10.1126/science.1151107 PMC2421010

[4] Ghosh R, Andersen EC, Shapiro JA, Gerke JP, Kruglyak L (2012) Natural variation in a chloride channel subunit confers avermectin resistance in C. elegans. Science 335: 574–578 doi:10.1126/science.1214318 22301316 10.1126/science.1214318 PMC3273849

[5] Bendesky A, Tsunozaki M, Rockman MV, Kruglyak L, Bargmann CI (2011) Catecholamine receptor polymorphisms affect decision-making in C. elegans. Nature 472: 313–318 doi:10.1038/nature09821 21412235 10.1038/nature09821 PMC3154120

[6] Palopoli MF, Rockman MV, TinMaung A, Ramsay C, Curwen S, et al (2008) Molecular basis of the copulatory plug polymorphism in Caenorhabditis elegans. Nature 454: 1019–1022 doi:10.1038/nature07171 18633349 10.1038/nature07171 PMC2597896

[7] McGrath PT, Rockman MV, Zimmer M, Jang H, Macosko EZ, et al (2009) Quantitative mapping of a digenic behavioral trait implicates globin variation in C. elegans sensory behaviors. Neuron 61: 692–699 doi:10.1016/j.neuron.2009.02.012 19285466 10.1016/j.neuron.2009.02.012 PMC2772867

[8] Reddy KC, Andersen EC, Kruglyak L, Kim DH (2009) A polymorphism in npr-1 is a behavioral determinant of pathogen susceptibility in C. elegans. Science 323: 382–384 doi:10.1126/science.1166527 19150845 10.1126/science.1166527 PMC2748219

[9] Glauser DA, Chen WC, Agin R, Macinnis BL, Hellman AB, et al (2011) Heat avoidance is regulated by transient receptor potential (TRP) channels and a neuropeptide signaling pathway in Caenorhabditis elegans. Genetics 188: 91–103 doi:10.1534/genetics.111.127100 21368276 10.1534/genetics.111.127100 PMC3120139

[10] Duveau F, Félix M-A (2012) Role of pleiotropy in the evolution of a cryptic developmental variation in Caenorhabditis elegans. Plos Biol 10: e1001230 doi:10.1371/journal.pbio.1001230 22235190 10.1371/journal.pbio.1001230 PMC3250502

[11] GAERTNER BE, Parmenter MD, Rockman MV, Kruglyak L, Phillips PC (2012) More than the sum of its parts: a complex epistatic network underlies natural variation in thermal preference behavior in Caenorhabditis elegans. Genetics 192: 1533–1542 doi:10.1534/genetics.112.142877 23086219 10.1534/genetics.112.142877 PMC3512158

[12] Bendesky A, Pitts J, Rockman MV, Chen WC, Tan M-W, et al (2012) Long-range regulatory polymorphisms affecting a GABA receptor constitute a quantitative trait locus (QTL) for social behavior in Caenorhabditis elegans. PLoS Genet 8: e1003157 doi:10.1371/journal.pgen.1003157 23284308 10.1371/journal.pgen.1003157 PMC3527333

[13] Kammenga JE, Doroszuk A, Riksen JAG, Hazendonk E, Spiridon L, et al (2007) A Caenorhabditis elegans wild type defies the temperature-size rule owing to a single nucleotide polymorphism in tra-3. PLoS Genet 3: e34 doi:10.1371/journal.pgen.0030034 17335351 10.1371/journal.pgen.0030034 PMC1808073

[14] Andersen EC, Gerke JP, Shapiro JA, Crissman JR, Ghosh R, et al (2012) Chromosome-scale selective sweeps shape Caenorhabditis elegans genomic diversity. Nat Genet 1–8 doi:10.1038/ng.1050 10.1038/ng.1050 PMC3365839 22286215

[15] Rockman MV, Kruglyak L (2009) Recombinational landscape and population genomics of Caenorhabditis elegans. PLoS Genet 5: e1000419 doi:10.1371/journal.pgen.1000419 19283065 10.1371/journal.pgen.1000419 PMC2652117

[16] GAERTNER BE, Phillips PC (2010) *Caenorhabditis elegans* as a platform for molecular quantitative genetics and the systems biology of natural variation. Genet Res 92: 331–348 doi:10.1017/S0016672310000601 10.1017/S0016672310000601 21429266

[17] Yvert G, Brem RB, Whittle J, Akey JM, Foss E, et al (2003) Trans-acting regulatory variation in Saccharomyces cerevisiae and the role of transcription factors. Nat Genet 35: 57–64 doi:10.1038/ng1222 12897782 10.1038/ng1222

[18] Brem RB, Yvert G, Clinton R, Kruglyak L (2002) Genetic dissection of transcriptional regulation in budding yeast. Science 296: 752–755 doi:10.1126/science.1069516 11923494 10.1126/science.1069516

[19] van Zanten M, Snoek LB, Proveniers MCG, Peeters AJM (2009) The many functions of ERECTA. Trends Plant Sci 14: 214–218 doi:10.1016/j.tplants.2009.01.010 19303350 10.1016/j.tplants.2009.01.010

[20] Rogers C, Reale V, Kim K, Chatwin H, Li C, et al (2003) Inhibition of Caenorhabditis elegans social feeding by FMRFamide-related peptide activation of NPR-1. Nat Neurosci 6: 1178–1185 doi:10.1038/nn1140 14555955 10.1038/nn1140

[21] de Bono M, Bargmann CI (1998) Natural variation in a neuropeptide Y receptor homolog modifies social behavior and food response in C. elegans. Cell 94: 679–689. 9741632 10.1016/s0092-8674(00)81609-8

[22] Macosko EZ, Pokala N, Feinberg EH, Chalasani SH, Butcher RA, et al (2009) A hub-and-spoke circuit drives pheromone attraction and social behaviour in C. elegans. Nature 458: 1171 doi:10.1038/nature07886 19349961 10.1038/nature07886 PMC2760495

[23] Reddy KC, Andersen EC, Kruglyak L, Kim DH (2009) A polymorphism in npr-1 is a behavioral determinant of pathogen susceptibility in C. elegans. Science 323: 382–384 doi:10.1126/science.1166527 19150845 10.1126/science.1166527 PMC2748219

[24] Chang AJ, Chronis N, Karow DS, Marletta MA, Bargmann CI (2006) A distributed chemosensory circuit for oxygen preference in C. elegans. Plos Biol 4: e274 doi:10.1371/journal.pbio.0040274 16903785 10.1371/journal.pbio.0040274 PMC1540710

[25] Davies AG, Bettinger JC, Thiele TR, Judy ME, McIntire SL (2004) Natural variation in the npr-1 gene modifies ethanol responses of wild strains of C. elegans. Neuron 42: 731–743 doi:10.1016/j.neuron.2004.05.004 15182714 10.1016/j.neuron.2004.05.004

[26] Jang H, Kim K, Neal SJ, Macosko E, Kim D, et al (2012) Neuromodulatory State and Sex Specify Alternative Behaviors through Antagonistic Synaptic Pathways in C. elegans. Neuron 75: 585–592 doi:10.1016/j.neuron.2012.06.034 22920251 10.1016/j.neuron.2012.06.034 PMC3462069

[27] Hallem EA, Sternberg PW (2008) Acute carbon dioxide avoidance in Caenorhabditis elegans. Proceedings of the National Academy of Sciences 105: 8038–8043 doi:10.1073/pnas.0707469105 10.1073/pnas.0707469105 PMC2430355 18524955

[28] Choi S, Chatzigeorgiou M, Taylor KP, Schafer WR, Kaplan JM (2013) Analysis of NPR-1 Reveals a Circuit Mechanism for Behavioral Quiescence in C. elegans. Neuron 78: 869–880 doi:10.1016/j.neuron.2013.04.002 23764289 10.1016/j.neuron.2013.04.002 PMC3683153

[29] Yook K, Harris TW, Bieri T, Cabunoc A, Chan J, et al (2012) WormBase 2012: more genomes, more data, new website. Nucleic Acids Res 40: D735–D741 doi:10.1093/nar/gkr954 22067452 10.1093/nar/gkr954 PMC3245152

[30] Kamath RS, Fraser AG, Dong Y, Poulin G, Durbin R, et al (2003) Systematic functional analysis of the Caenorhabditis elegans genome using RNAi. Nature 421: 231–237 doi:10.1038/nature01278 12529635 10.1038/nature01278

[31] Cheung BHH, Arellano-Carbajal F, Rybicki I, de Bono M (2004) Soluble guanylate cyclases act in neurons exposed to the body fluid to promote C. elegans aggregation behavior. Curr Biol 14: 1105–1111 doi:10.1016/j.cub.2004.06.027 15203005 10.1016/j.cub.2004.06.027

[32] Gray JM, Karow DS, Lu H, Chang AJ, Chang JS, et al (2004) Oxygen sensation and social feeding mediated by a C. elegans guanylate cyclase homologue. Nature 430: 317–322 doi:10.1038/nature02714 15220933 10.1038/nature02714

[33] Rockman MV, Kruglyak L (2006) Genetics of global gene expression. Nat Rev Genet 7: 862–872 doi:10.1038/nrg1964 17047685 10.1038/nrg1964

[34] Seidel HS, Kimble J (2011) The oogenic germline starvation response in C. elegans. PLoS ONE 6: e28074 doi:10.1371/journal.pone.0028074 22164230 10.1371/journal.pone.0028074 PMC3229504

[35] Jo H, Shim J, Lee JH, Lee J, Kim JB (2009) IRE-1 and HSP-4 contribute to energy homeostasis via fasting-induced lipases in C. elegans. Cell Metab 9: 440–448 doi:10.1016/j.cmet.2009.04.004 19416714 10.1016/j.cmet.2009.04.004

[36] Avery L, Horvitz HR (1990) Effects of starvation and neuroactive drugs on feeding in Caenorhabditis elegans. J Exp Zool 253: 263–270 doi:10.1002/jez.1402530305 2181052 10.1002/jez.1402530305

[37] Luedtke S, O'Connor V, Holden-Dye L, Walker RJ (2010) The regulation of feeding and metabolism in response to food deprivation in Caenorhabditis elegans. Invert Neurosci 10: 63–76 doi:10.1007/s10158-010-0112-z 21120572 10.1007/s10158-010-0112-z

[38] Hubbard EJA, Korta DZ, Dalfó D (2013) Physiological control of germline development. Adv Exp Med Biol 757: 101–131 doi:_10.1007/978-1-4614-4015-4_5 22872476 10.1007/978-1-4614-4015-4_5 PMC3760422

[39] Dalfó D, Michaelson D, Hubbard EJA (2012) Sensory regulation of the C. elegans germline through TGF-β-dependent signaling in the niche. Curr Biol 22: 712–719 doi:10.1016/j.cub.2012.02.064 22483938 10.1016/j.cub.2012.02.064 PMC3633564

[40] Gloria-Soria A, Azevedo RBR (2008) npr-1 Regulates foraging and dispersal strategies in Caenorhabditis elegans. Curr Biol 18: 1694–1699 doi:10.1016/j.cub.2008.09.043 18993077 10.1016/j.cub.2008.09.043

[41] Torii KU, Mitsukawa N, Oosumi T, Matsuura Y, Yokoyama R, et al (1996) The Arabidopsis ERECTA gene encodes a putative receptor protein kinase with extracellular leucine-rich repeats. Plant Cell 8: 735–746 doi:10.1105/tpc.8.4.735 8624444 10.1105/tpc.8.4.735 PMC161133

[42] Weber KP, De S, Kozarewa I, Turner DJ, Babu MM, et al (2010) Whole genome sequencing highlights genetic changes associated with laboratory domestication of C. elegans. PLoS ONE 5: e13922 doi:10.1371/journal.pone.0013922 21085631 10.1371/journal.pone.0013922 PMC2978686

[43] McGrath PT, Xu Y, Ailion M, Garrison JL, Butcher RA, et al (2011) Parallel evolution of domesticated Caenorhabditis species targets pheromone receptor genes. Nature 477: 321–325 doi:10.1038/nature10378 21849976 10.1038/nature10378 PMC3257054

[44] Rockman MV (2011) THE QTN PROGRAM AND THE ALLELES THAT MATTER FOR EVOLUTION: ALL THAT'S GOLD DOES NOT GLITTER. Evolution no–no doi:10.1111/j.1558-5646.2011.01486.x 10.1111/j.1558-5646.2011.01486.x PMC3386609 22220860

[45] Broman KW, Wu H, Sen S, Churchill GA (2003) R/qtl: QTL mapping in experimental crosses. Bioinformatics 19: 889–890. 12724300 10.1093/bioinformatics/btg112

[46] Lander E, Botstein D (1989) Mapping Mendelian Factors Underlying Quantitative Traits Using RFLP Linkage Maps. Genetics 121: 185. 2563713 10.1093/genetics/121.1.185 PMC1203601

[47] Doerge RW, Churchill GA (1996) Permutation tests for multiple loci affecting a quantitative character. Genetics 142: 285–294. 8770605 10.1093/genetics/142.1.285 PMC1206957

[48] Haley CS, Knott SA (1992) A simple regression method for mapping quantitative trait loci in line crosses using flanking markers. Heredity 69: 315–324. 16718932 10.1038/hdy.1992.131

[49] Bloom JS, Ehrenreich IM, Loo WT, Lite T-LV, Kruglyak L (2013) Finding the sources of missing heritability in a yeast cross. Nature 494: 234–237 doi:10.1038/nature11867 23376951 10.1038/nature11867 PMC4001867

[50] Brem RB, Kruglyak L (2005) The landscape of genetic complexity across 5,700 gene expression traits in yeast. Proc Natl Acad Sci USA 102: 1572–1577 doi:10.1073/pnas.0408709102 15659551 10.1073/pnas.0408709102 PMC547855

[51] Smyth GK (2005) Limma: linear models for microarray data. Bioinformatics and Computational Biology Solutions Using R and Bioconductor Statistics for Biology and Health 397–420.

[52] Capra EJ, Skrovanek SM, Kruglyak L (2008) Comparative developmental expression profiling of two C. elegans isolates. PLoS ONE 3: e4055 doi:10.1371/journal.pone.0004055 19116648 10.1371/journal.pone.0004055 PMC2605249

[53] Antonov AV, Schmidt T, Wang Y, Mewes HW (2008) ProfCom: a web tool for profiling the complex functionality of gene groups identified from high-throughput data. Nucleic Acids Res 36: W347–W351 doi:10.1093/nar/gkn239 18460543 10.1093/nar/gkn239 PMC2447768

