# Correction: A Variant in the Neuropeptide Receptor *npr-1* is a Major Determinant of *Caenorhabditis elegans* Growth and Physiology

**DOI:** 10.1371/journal.pgen.1004316

**Published:** 2014-03-27

**Authors:** The PLOS Genetics Staff

There is an error in the third sentence of the Author Summary. The correct sentence is:

Moreover, we found that variation in *npr-1* is also responsible for differences in 247 gene expression traits.

There is also an error in formatting of the legend of Figure 3. The complete, correct Figure 3 legend is:

**Figure 3:**
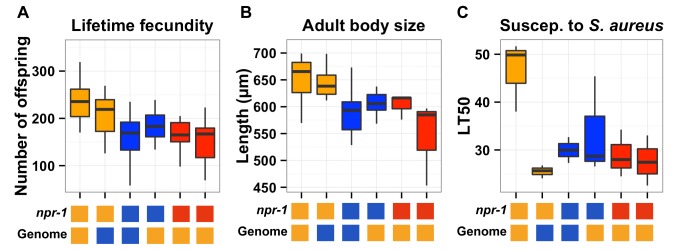
Phenotypic distributions of three quantitative traits for the parents, nearly isogenic lines, and *npr-1* mutants. Box plots show the summary phenotype data of the two parents (Bristol in orange and Hawaii in blue), the two nearly isogenic lines (*kyIR9* in orange and *qgIR1*in blue), and the two independent *npr-1* loss-of-function alleles (*npr-1(ad609)* and *npr-1(ky13)* in red). From left to right, the lifetime fecundity (**A**), the mean length of adult animals (**B**), and the LT50 distribution after exposure to *S. aureus* (**C**). Below each plot are two boxes. The top box denotes the *npr-1* genotype: laboratory-derived NPR-1V from Bristol in orange, ancestral wild-type NPR-1F from Hawaii in blue, or loss-of-function allele from Bristol in red. The bottom box denotes the genome-wide genotype: Bristol in orange and Hawaii in blue. Statistical significance was tested using Tukey's HSD. For (**A**), the *qgIR1* strain has significantly fewer offspring than the Bristol strain (p  =  0.003) does, and the *kyIR9* strain has significantly more offspring than the Hawaii strain (p  =  0.0059) does. The Hawaii strain, *qgIR1,* and the two *npr-1* loss-of-function alleles do not have significantly different numbers of offspring. The same is true for the Bristol strain and *kyIR9*. For (**B**), the *qgIR1* strain is significantly smaller than the Bristol strain (p  =  0.00148), and the *kyIR9* strain is significantly larger than the Hawaii strain (p  =  6E-5). The Hawaii strain, *qgIR1*, and the two *npr-1* loss-of-function alleles do not have significantly different lengths. The same is true for the Bristol strain and *kyIR9* introgression strain.
