# Supplementary figures and images for: A Variant in the Neuropeptide Receptor npr-1 is a Major Determinant of Caenorhabditis elegans Growth and Physiology
Source: PLoS Genet. 2014 Feb 27;10(2):e1004156. doi: 10.1371/journal.pgen.1004156 (PMC3937155; doi:10.1371/journal.pgen.1004156)

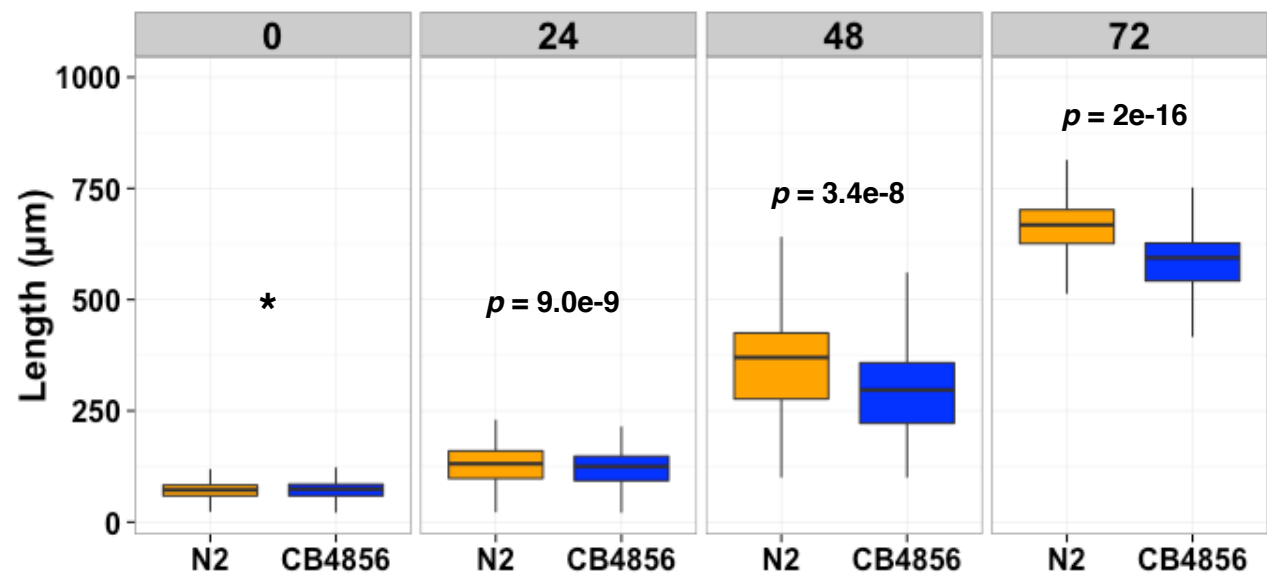

Supplement: Figure S1 — We observed that the Bristol strain (orange) is larger than the Hawaii strain (blue) at all-time points throughout C. elegans development, except for the zero hour arrested L1 time point. Size differences of L1 larvae are likely determined largely by maternal effects. In three independent assays, we observed examples of both the Bristol strain being smaller and larger than the Hawaii strain. For this reason, we indicated the uncertainty with an asterisk. The p-value for each comparison (Tukey's HSD) is shown above the boxplots for each time point. (PDF) [file pgen.1004156.s001.pdf]

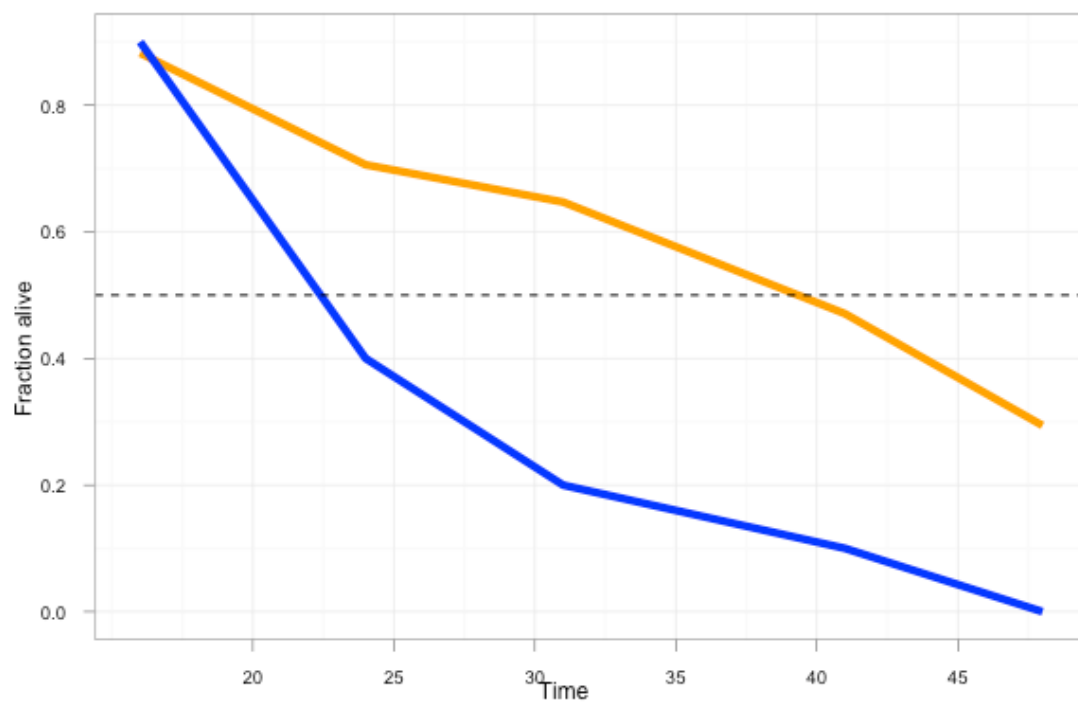

Supplement: Figure S2 — An example survival curve of Bristol (orange) and Hawaii (blue) after exposure to the opportunistic human pathogen S. aureus. The dotted line denotes when half of the population was alive or dead. (PDF) [file pgen.1004156.s002.pdf]
